# The Effectiveness of Four Nicotinamide Adenine Dinucleotide (NAD^+^) Precursors in Alleviating the High-Glucose-Induced Damage to Hepatocytes in *Megalobrama amblycephala*: Evidence in NAD^+^ Homeostasis, Sirt1/3 Activation, Redox Defense, Inflammatory Response, Apoptosis, and Glucose Metabolism

**DOI:** 10.3390/antiox13040385

**Published:** 2024-03-22

**Authors:** Yanzou Dong, Xi Wang, Luyao Wei, Zishang Liu, Xiaoyu Chu, Wei Xiong, Wenbin Liu, Xiangfei Li

**Affiliations:** Key Laboratory of Aquatic Nutrition and Feed Science of Jiangsu Province, College of Animal Science and Technology, Nanjing Agricultural University, No. 1 Weigang Road, Nanjing 210095, China

**Keywords:** NAD^+^ precursors, hepatocyte health, oxidative stress, apoptosis, glucose metabolism

## Abstract

The administration of NAD^+^ precursors is a potential approach to protect against liver damage and metabolic dysfunction. However, the effectiveness of different NAD^+^ precursors in alleviating metabolic disorders is still poorly elucidated. The current study was performed to compare the effectiveness of four different NAD^+^ precursors, including nicotinic acid (NA), niacinamide (NAM), nicotinamide riboside (NR), and nicotinamide mononucleotide (NMN) in alleviating high-glucose-induced injury to hepatocytes in a fish model, *Megalobrama amblycephala*. An in vitro high-glucose model was successfully established to mimic hyperglycemia-induced damage to the liver, which was evidenced by the reduced cell viability, the increased transaminase activity, and the depletion of cellular NAD^+^ concentration. The NAD^+^ precursors all improved cell viability, with the maximal effect observed in NR, which also had the most potent NAD^+^ boosting capacity and a significant Sirt1/3 activation effect. Meanwhile, NR presented distinct and superior effects in terms of anti-oxidative stress, inflammation inhibition, and anti-apoptosis compared with NA, NAM, and NMN. Furthermore, NR could effectively benefit glucose metabolism by activating glucose transportation, glycolysis, glycogen synthesis and the pentose phosphate pathway, as well as inhibiting gluconeogenesis. Moreover, an oral gavage test confirmed that NR presented the most potent effect in increasing hepatic NAD^+^ content and the NAD^+^/NADH ratio among four NAD^+^ precursors. Together, the present study results demonstrated that NR is most effective in attenuating the high-glucose-induced injury to hepatocytes in fish compared to other NAD^+^ precursors.

## 1. Introduction

For a number of years, metabolic diseases, including diabetes, obesity and non-alcoholic fatty liver disease (NAFLD) have been prevalent in both developed and developing countries, becoming a strong public health concern [1,2,3]. Abnormal hyperglycemia is the characteristic and distinct pathologic feature of diabetes, and it has also been frequently found in patients suffering obesity or/and NAFLD [3,4]. Longstanding high blood glucose has been found to be associated with a cascade of adverse health consequences. Among them, high-glucose-induced liver damage often worsens metabolic dysfunction and contributes to more severe and irreversible pathological consequences such as non-alcoholic steatohepatitis, cirrhosis, and even cancers [3]. Although the exact mechanism is not completely understood, oxidative stress has been reported to contribute to high-glucose-mediated injury to organs [5]. Chronic hyperglycemia could break the redox equilibrium. Consequently, the accumulated reactive oxygen species (ROS) could accelerate liver damage by activating the inflammatory response and the apoptosis pathway [6,7]. This process further exacerbates insulin resistance and glucose metabolism dysfunction, thereby forming a vicious cycle [8,9].

Nicotinamide adenine dinucleotide (NAD^+^) is one of the critical biomolecules for cellular redox balance. As an enzyme cofactor/co-substrate, NAD^+^ participates in many critical physiological processes, including protein deacylation, DNA repair, inflammatory reactions, and defense against oxidative stress [10]. Previous studies in mammals have shown that the DNA damage induced by aberrant nutritional status exaggerates cellular NAD^+^ consumption. Declines in NAD^+^ levels consequently cause oxidative stress and contribute to the pathological processes of metabolic diseases [10,11]. The above facts suggest that rescuing NAD^+^ deficiency is a potential approach to protect hepatocytes against damage induced by high glucose. Due to its high bioactivity, it is extremely difficult to directly provide exogenous NAD^+^ to animals [11,12]. Vitamin B_3_ (also known as nicotinic acid, NA) and niacinamide (NAM), as well as their derivatives like nicotinamide riboside (NR) and nicotinamide mononucleotide (NMN), are well-described NAD^+^ precursor vitamins [13,14]. The administration of these NAD^+^ donors could boost cellular NAD^+^ content, thereby alleviating metabolic dysfunctions. It is noteworthy that emerging evidence has proposed different pharmacokinetics and functions of these NAD^+^ precursors, which could be due to their diverse metabolic properties and biochemical characteristics [15]. Specifically, a previous study has shown that NA, NR, and NMN differ in the degree to which they improve NAD^+^ concentration in several mammalian cell lines [11]. Moreover, a more recent study has demonstrated that the oral administration of NR displays a stronger ability to enhance hepatic NAD^+^ concentration than NA or NAM in mice [16]. Despite this, to the best of our knowledge, no study is available comparing the effectiveness of different NAD^+^ precursors in alleviating metabolic dysfunctions.

Considering that the related metabolic pathways are well conserved among different animal species, studies using lower animals are deemed to be able to provide new insights into the understanding of metabolism diseases in human counterparts [17]. Indeed, previous studies have revealed the similar pathophysiological pathways behind the metabolic disease in mammal and fish models [18,19,20]. Considering this fact, fish are deemed to be convenient, reliable, and low-cost experimental animals to substitute for mammals [21,22]. Blunt snout bream (*Megalobrama amblycephala*) is a kind of cyprinid fish with high commercial importance [18]. Due to its herbivorous feeding habit, this species is prone to the high-glucose-induced hepatocytes injury caused by the feeding of high-carbohydrate diets [23]. NAD^+^ depletion has also notably been found in individuals this species suffering from hyperglycemia [24]. Thus, *M. amblycephala* is a suitable subject to study high-glucose-induced liver damage. Based on the above facts, the present study was conducted to compare the effectiveness of four different NAD^+^ precursors in alleviating high-glucose-induced hepatocytes damage in *M. amblycephala*. To fulfill this goal, cell viability, transaminase activity, NAD^+^ homeostasis, Sirt1/3 activation, redox equilibrium, inflammatory response, apoptosis, and glucose metabolism were all investigated. The results obtained may promote the development of effective nutritional interventions to alleviate metabolic dysfunctions in animals and humans.

## 2. Materials and Methods

### 2.1. Primary Hepatocytes Isolation and Culture

The isolation and culture procedures of primary *M. amblycephala* hepatocytes were carried out with reference to the published protocols [25] with slight modifications. Briefly, fish (body weight = 20.5 ± 0.5 g) were kept in freshwater, with a 1% streptomycin and penicillin solution added, and fasted overnight. Next, fish were anesthetized using MS-222 (100 mg/L, tricaine methanesulfonate, Sigma, St. Louis, MO, USA) and bled by cutting the gill arches. The liver was dissected under aseptic conditions, washed with a pH 7.0 phosphate buffer twice, and cut into 1 mm^3^ pieces on an ice bed. Then, the pieces were digested with a 0.25% trypsin-EDTA solution at 28 °C for 40 min. The cell suspension was obtained after filtration through a 200 mesh cell strainer and centrifugation at 300× *g* for 5 min. The harvested hepatocytes were transferred to a cell culture plate and maintained in the DMEM/F12 medium supplemented with 10% fetal bovine serum and 2% streptomycin and penicillin (termed as complete medium) at 28 °C in a humidified atmosphere with 5% CO_2_.

### 2.2. Cell Treatment

The adherent primary hepatocytes were serum-starved overnight after reaching 80% confluency. Then, the cell culture media were replaced with fresh media for further experiments. Cells cultured with a normal complete medium were used as the control group. The high-glucose medium was prepared by adding D-glucose to the complete medium, thereby obtaining the high-glucose group. Furthermore, four different NAD^+^ precursors, namely nicotinic acid (NA), niacinamide (NAM), nicotinamide riboside (NR), and nicotinamide mononucleotide (NMN), were added to the high-glucose medium at doses of 10 and 50 μM, thereby obtaining the NAD^+^-precursor treatment groups. The supplementation levels of the NAD^+^ precursors were determined based on the results of our preliminary test, which obtained the highest cell viability at the dose of 50 μM.

### 2.3. Cell Viability

Primary hepatocytes were seeded into 24 well plates at a density of 1 × 10^6^ cells per well. After treatments, cells were washed with a phosphate buffer and then induced with the DMEM/F12 medium containing 10% Cell Counting Kit-8 regent (CCK8, ApexBio Technology, Shanghai, China) for 2 h. Then, the absorbance was measured at a 450 nm wavelength. The value of the relative cell viability was calculated by normalizing to the control group.

### 2.4. Transaminase Activity Detection

The collected cell culture mediums were centrifugated at 800× *g* for 5 min with the harvested supernatants taken to measure the alanine aminotransferase (ALT) and aspartate transaminase (AST) activities using an enzymatic colorimetric method according to the published protocol [26]. Briefly, the sample was incubated with an a-Ketoglutarate buffer containing L-alanine (for ALT detection) or L-aspartate (for AST detection) at 37 °C for 60 min. Then, 2, 4-DNPH was added to the reaction systems, and the mixture was allowed to stand at room temperature for 20 min. After being mixed with 0.4 M NaOH solution and incubated for 10 min, the absorbance was measured at 505 nm.

### 2.5. Detection of NAD^+^ and NADH Contents

The NAD^+^ and NADH concentrations were determined based on the WST-8-dependent chromogenic reaction according to the published protocols [27]. The amounts of total NAD(H) or NADH in the samples were measured, and the NAD^+^ amount was calculated by subtracting the NADH amount from that of total NAD(H). For total NAD(H) determination, the sample was mixed with a dehydrogenase working solution and incubated at 37 °C for 10 min. Next, a reaction buffer (20 mM Tris-HCl, 200 μM WST-8, and 8 μM PMS, pH 9.0) was added, and the reaction system was kept at 37 °C for another 30 min. Then, the absorbance was measured at 450 nm. For NADH measurement, the sample was first heating at 60 °C for 10 min. Then, the same procedure was performed again.

### 2.6. Antioxidant Status Examination

The hepatocytes were digested using a 0.25% trypsin-EDTA solution after different treatments. The harvested cells were homogenized in a phosphate buffer. Then, the cellular malonaldehyde (MDA) content was determined through the thiobarbituric acid (TBA) test based on published protocols [28]. Briefly, the sample was mixed with 20% trichloroacetic acid (TCA) and then dispersed in 0.05 M H_2_SO_4_ solution. Next, TBA (0.2% in sodium sulphate) and N-butanol were added to the mixture, which was subsequently heated at 100 °C for 1 h. After cooling on ice, the absorbance was measured at 530 nm.

The activities of antioxidant enzymes, including superoxide dismutase (SOD), catalase (CAT), and glutathione peroxidase (GPX), were all assayed using the methods described previously [29,30]. For the determination of SOD activity, the sample was added to the reaction system containing xanthine, WST-1, and Formosan dye. After being kept at 37 °C for 30 min, the absorbance of the reaction system was obtained at 450 nm. For the CAT activity assay, the sample was mixed with the reaction buffer (1% Triton X-100, 30% H_2_O_2_) and kept at 37 °C for 1 min. Then, the ammonium molybdate solution was added and the absorbance was measured at 405 nm. For GPX activity measurement, the sample was added to the reaction mixture containing 1 U glutathione reductase, 2 mM glutathione, 0.12 mM NADPH, 2 mM H_2_O_2_, and 6 mM cumene hydroperoxide and was incubated at 37 °C for 1 min. Then, dithiodinitrobenzoic acid was used to visualize the product in a color reaction, and the absorbance was measured at 412 nm.

### 2.7. Measurement of Inflammatory Cytokines

The concentrations of inflammatory cytokines including interleukin-6 (H007-1, IL6), interleukin-1β (H002-1, IL1β), and tumor necrosis factor-α (H052-1, TNFα) in the cell culture supernatants were all evaluated by cytokine-specific enzyme-linked immunosorbent assay (ELISA) kits according to the instructions provided by the manufacturer (Nanjing Jiancheng Institute of Bioengineering, Nanjing, China). Briefly, the sample was transferred to plates coated by specific antibodies labeled with horseradish peroxidase and was kept at 37 °C for 1 h. Next, the plates were washed 5 times with phosphate buffer containing 0.05% Tween-20. The chromogenic substrate solutions A and B were added to the plates to develop color. The absorbance was measured at 450 nm.

### 2.8. Caspase 3 Activity Assay

For the measurement of caspase 3 activity, the hepatocytes were washed with a phosphate buffer and were collected by a lysate buffer (10 mM PIPES, 2 mM EDTA, 1% NP40 and 4 mM DTT, pH 7.5) after treatments. Then, the cell lysates were centrifugated at 16,000× *g* for 15 min, and the obtained supernatants were incubated with 200 μmol of caspase 3 substrate Ac-DEVD-pNA (acetyl-Asp-Glu-Val-Asp p-nitroanilide) at 37 °C for 10 h. The absorbance was quantified at a wavelength of 405 nm.

### 2.9. Glucose Consumption Test

To test the glucose consumption of cells, primary hepatocytes were seeded into 24-well plates at a density of 1 × 10^6^ cells per well. After the above-mentioned treatments, the cell culture supernatants were collected and used for the glucose concentration assay. At the same time, cells were digested using a 0.25% trypsin-EDTA solution and homogenized in a phosphate buffer for the quantification of cellular proteins. The media of each group collected before the experimental treatment were used to determine the initial glucose concentrations. Thereafter, the variations of glucose amounts in the cell culture medium during the treatments were calculated, and the values were normalized to the cellular protein contents. The glucose concentrations were then measured by the glucose oxidase method [31].

### 2.10. Glucose Production Assay

For cellular glucose production assay, cells after different treatments were rinsed with phosphate buffer twice and were inducted in glucose-free DMEM/F12 medium (Procell Life Science & Technology, Wuhan, China). After 6 h, the cell culture supernatants were collected and used for the glucose concentration test. The results were normalized by the protein concentration.

### 2.11. Glycogen Determination

For glycogen measurement, the hepatocytes were harvested through 0.25% trypsin-EDTA solution digestion and then subjected to base hydrolysis. Then, the hydrolysate was heated in a boiling water bath for 20 min. After being cooled down by flowing water, the reaction solution was incubated with a reaction mixture containing 50% sulfuric acid and 5 mM anthrone at 100 °C for 5 min. The absorbance was quantified at a wavelength of 620 nm after cooling.

### 2.12. Gene Expression Analysis

The primary hepatocytes were lysed using the AG RNAex Pro regent (Accurate Biotechnology (Hunan) Co., Ltd., Changsha, China) for 5 min at room temperature. Then, total RNA was extracted from the lysis using a SteadyPure RNA Extraction Kit with reference to the protocols provided by Accurate Biotechnology. Agarose gel electrophoresis was performed to confirm the quality of RNA samples with the purity determined by a Nanodrop UV spectrophotometer (Thermo Fisher Scientific, Waltham, MA, USA). Then, the first-strand complementary DNA (cDNA) was obtained by reverse transcription from 1 μg of total RNA using an Evo M-MLV RT Kit (Accurate Biotechnology (Hunan) Co., Ltd., Changsha, China). To quantify gene expression, the real-time PCR (RT-PCR) was conducted under the Bio-Rad CFX96 platform (Bio-Rad, Berkeley, CA, USA) using the SYBR Green Pro Taq HS premix acquired from Accurate Biotechnology. The 2^−∆∆Ct^ method was adopted to calculate the relative transcription with elongation factor 1α (*ef1a*) used as the housekeeping gene. The high expression stability of *ef1a* in *M. amblycephala* was demonstrated in a recent study [32]. Moreover, the sequences of primers used in the current study are listed in Table 1.

### 2.13. Animal Experiment

In order to compare the effects of four NAD^+^ precursors (NA, NAM, NR, and MNM) on the hepatic NAD^+^ concentration at the in vivo level, an animal experiment was performed. *M. amblycephala* (body weight = 82.5 ± 1.5 g) were purchased from a national aquaculture hatchery located at Ezhou, Hubei province, China. All fish shared a consistent genetic background. Before the test, fish were transported to a recirculating aquaculture system for one week for acclimation. During this period, a commercial diet (containing 30% protein, 5% lipids, and 38% carbohydrates) was fed to the fish twice daily (8:30 and 17:30 h). After that, fish were fasted for 24 h and were slightly euthanized using a 50 mg/L tricaine methanesulfonate (MS-222, Sigma, USA) solution to reduce the stress response. Then, a total of 60 experimental fish were randomly divided into 5 groups with an oral administration of normal saline (5 mL/kg body weight, the vehicle group) and different NAD^+^ precursors (NA, NAM, NR, and NMN, 50 μmol/kg body weight). The dose of NAD^+^ precursors was determined based on the five-fold molar amount of the optimal daily dietary NA amount of *M. amblycephala* [33]. Three sampling time points were set at 1, 3, and 12 h after the gavage test referring to a previous study [16], and four fish forming each group were sampled at each sampling time point. Fish were euthanized using a 100 mg/L MS-222 solution before sampling and then were quickly dissected on an ice bed. The liver samples were collected, snap-frozen in liquid nitrogen, and transferred to −80 °C till use. Next, the hepatic NAD^+^ and NADH concentrations were measured according to the above-mentioned method. Meanwhile, the water parameters were monitored during this test and maintained as follows: pH—7.2 to 7.4; dissolved oxygen—about 5 mg/L; and ammonia nitrogen—<0.2 mg/L. The animal study was approved by the Animal Care and Use Committee of Nanjing Agricultural University (SYXK (Su) 2011-0036, Nanjing, China). Fish were kept under a suitable water environment, fed a nutrient-balanced feed, and sacrificed after being euthanized following standard ethical procedures. All these efforts were conducted to reduce the stress response of fish and ensure the welfare of fish.

### 2.14. Western Blot

The liver sample was lysed in RIPA buffer, mixed with loading buffer, and boiled for 5 min. Then, the sample was subjected to SDS-PAGE electrophoresis. The gel and electrophoresis solution contained 0.05% SDS. The separated proteins were transferred to polyvinylidene fluoride membranes. Subsequently, the membrane was blocked with 5% (*w*/*v*) non-fat dry milk and was then incubated with the first antibody and the second antibody successively. Then, bands were visualized by an electro-chemiluminescence system. The primary antibodies of β-actin (20536-1-AP, Proteintech), Sirtuin 1 (SIRT1) (13161-1-AP, Proteintech), and SIRT3 (10099-1-AP, Proteintech) were used in the present study.

### 2.15. Statistical Analysis

In the present study, all tests were performed in quadruplicate, and the results are shown as means ± standard error (SE). Statistical analyses were carried out using the SPSS 20.0 software. For the in vitro experiment, comparisons between the control and the high-glucose group were conducted using the Student’s *t*-test, while one-way ANOVA followed by the Tukey’s post hoc test was performed to identify the differences among the high-glucose group and the groups supplemented with NAD^+^ precursors. For the in vivo experiment, a two-way ANOVA test was used to analyze the differences among treatments means based on oral gavage, sampling times, and their interaction. Differences were considered statistically significant when *p* values were lower than 0.05.

## 3. Results

### 3.1. Establishment of the High-Glucose Model

**Figure 1:**
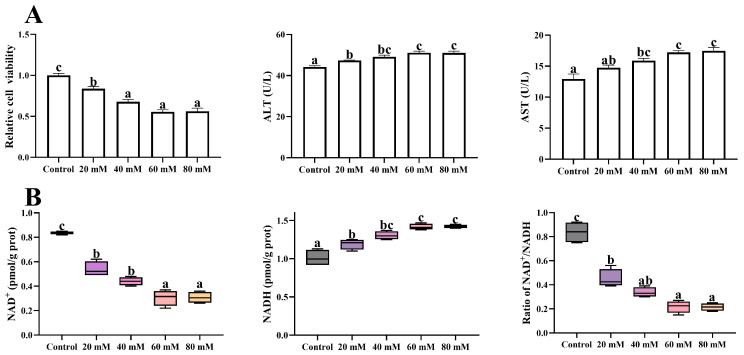
Hepatocellular damage indices (**A**) and NAD^+^ homeostasis (**B**) of the primary hepatocytes exposed to high-glucose media (additional 20, 40, 60, and 80 mM glucose) for 48 h. Data are presented as mean ± SE. Data (*n* = 4) marked with different letters mean significant differences (*p* < 0.05, Tukey’s test).

To set up the high-glucose model, high-glucose cell culture media were prepared by additionally adding glucose to the complete medium at the concentrations of 20, 40, 60, and 80 mM. These high-glucose media were used to incubate the primary hepatocytes for 48 h. The 40, 60, and 80 mM supplemented glucose treatments markedly declined the cell viability to 67.8, 55.4, and 56.1% of the control group (*p* < 0.05), respectively. Meanwhile, the ALT and AST activities decreased significantly when glucose concentration increased up to 60 mM (*p* < 0.05) (Figure 1A). As shown in Figure 1B, the lowest cellular NAD^+^ concentration and the NAD^+^/NADH ratio, as well as the highest NADH contents, were all observed in the 60 and 80 mM glucose treatments (*p* < 0.05). Based on the above results, the glucose concentration in the high-glucose group was set at 60 mM in the following tests.

### 3.2. NAD^+^ Precursors Improved the High-Glucose-Induced Hepatocyte Injury

**Figure 2:**
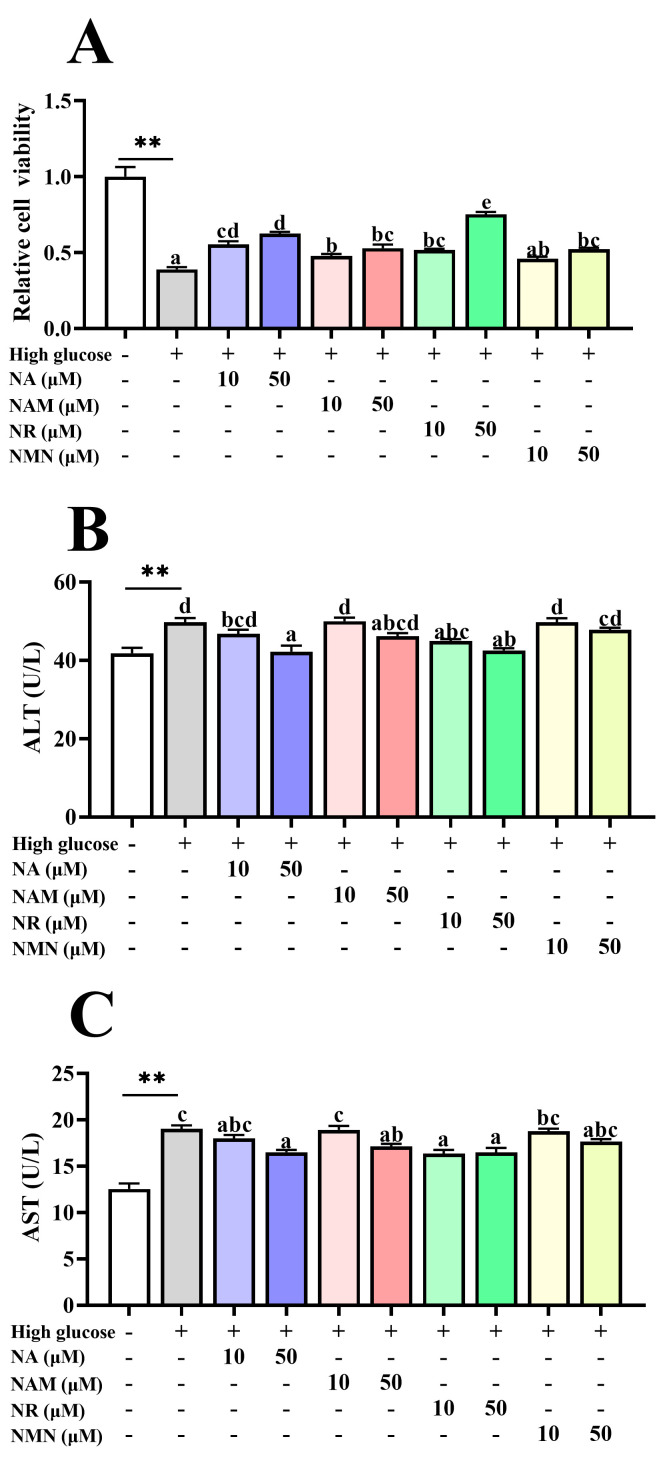
Effects of different NAD^+^ precursors on the hepatocellular damage indices (**A**–**C**) of primary hepatocytes exposed to a high-glucose medium (additional 60 mM glucose) for 48 h. Data (*n* = 4) are presented as mean ± SE. The asterisk (*) means a significant difference between the control and the high-glucose group (** *p* < 0.01, *t*-test). Different letters mean significant differences among the high-glucose group and the groups supplemented with NAD^+^ precursors (*p* < 0.05, Tukey’s test).

As shown in Figure 2A, the cell viability of the high-glucose group was markedly decreased compared to the control (*p* < 0.05). The NA, NAM, and NR addition at the doses of 10 and 50 μM significantly increased the cell viability of hepatocytes exposed to high glucose (*p* < 0.05), with the 50 μM NR group exhibiting the highest value. The NMN administration presented similar effects, but the significance could only be observed at the dose of 50 μM (*p* < 0.05). Furthermore, the high-glucose group had markedly higher ALT and AST activities than the control group in cell culture supernatants (*p* < 0.05), while the addition of 50 μM NA and 10 and 50 μM NR significantly alleviated it (*p* < 0.05) (Figure 2B,C).

### 3.3. NR Exhibited Superior NAD^+^ Boosting and Sirt 1/3 Activation Effects

**Figure 3:**
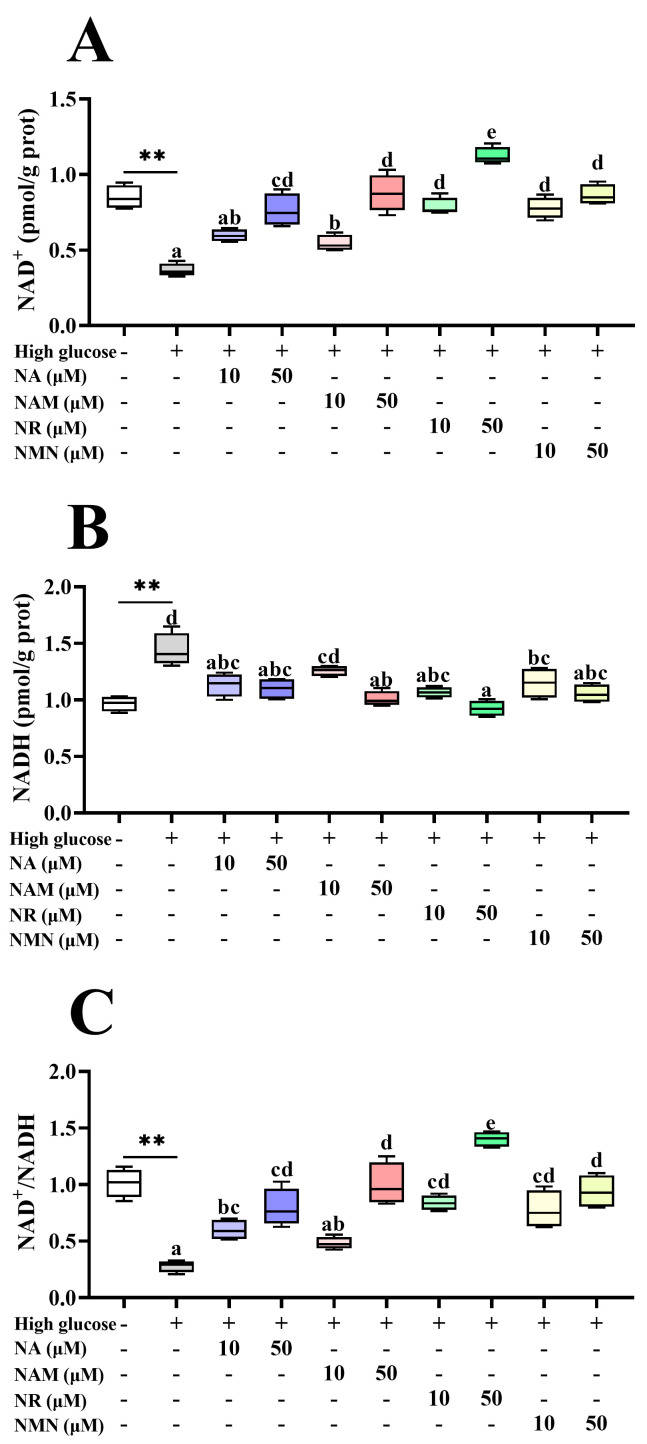
Effects of different NAD^+^ precursors on the NAD^+^ homeostasis (**A**–**C**) of primary hepatocytes exposed to a high-glucose medium (additional 60 mM glucose) for 48 h. Data (*n* = 4) are presented as mean ± SE. The asterisk (*) means a significant difference between the control and the high-glucose group (** *p* < 0.01, *t*-test). Different letters mean significant differences among the high-glucose group and the groups supplemented with NAD^+^ precursors (*p* < 0.05, Tukey’s test).

**Figure 4:**
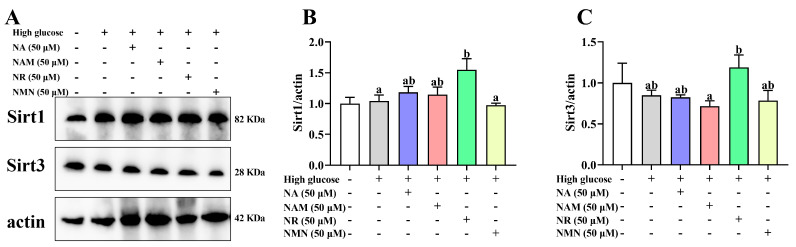
Effects of different NAD^+^ precursors on the Sirt1 and Sirt3 protein abundance of primary hepatocytes exposed to a high-glucose medium (additional 60 mM glucose) for 48 h. (**A**) Image of bands, (**B**) relative level of Sirt1 abundance, (**C**) relative level of Sirt3 abundance. Data (*n* = 4) are presented as mean ± SE. Different letters mean significant differences among the high-glucose group and the group supplemented with NAD^+^ precursors (*p* < 0.05, Tukey’s test).

As shown in Figure 3, compared with the control, the high-glucose treatment markedly decreased the NAD^+^ content and the NAD^+^/NADH ratio but increased the NADH content. All groups supplemented with NAD^+^ precursors had increased cellular NAD^+^ levels and the NAD^+^/NADH ratio with the highest value was noted in the 50 μM NR group (*p* < 0.05). Furthermore, all groups supplemented with NAD^+^ precursors had a significantly lower NADH content compared to the high-glucose group, except the 10 μM NAM group (*p* < 0.05). As shown in Figure 4, the Sirt1 protein expression in the NR-treated groups was significantly higher than the high-glucose group and the NMN treatment (*p* < 0.05) but showed no statistical difference with the NA and NAM groups (*p* > 0.05). The highest abundance of Sirt3 was also noted in the NR group, but the significance was only found between the NR and NAM groups (*p* < 0.05).

### 3.4. NAD^+^ Precursors Attenuated High-Glucose-Induced Oxidative Stress

**Figure 5:**
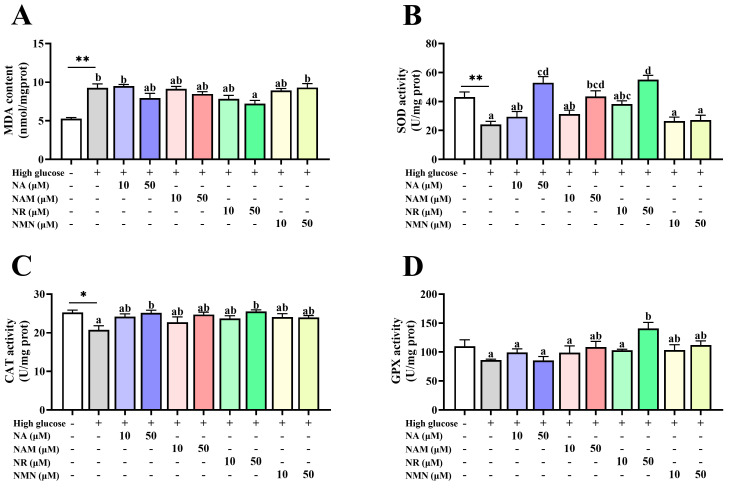
Effects of different NAD^+^ precursors on the antioxidant status (**A**–**D**) of primary hepatocytes exposed to a high-glucose medium (additional 60 mM glucose) for 48 h. Data (*n* = 4) are presented as mean ± SE. The asterisk (*) means a significant difference between the control and the high-glucose group (* *p* < 0.05, ** *p* < 0.01, *t*-test). Different letters mean significant differences among the high-glucose group and the groups supplemented with NAD^+^ precursors (*p* < 0.05, Tukey’s test).

As shown in Figure 5, compared with the control, the high-glucose incubation markedly increased the MDA content but decreased the CAT and SOD activities (*p* < 0.05). The supplementation of 50 μM NR in the high-glucose medium markedly reduced the MDA content but promoted the GPX activity (*p* < 0.05). The supplementation of NA, NAM, and NR at 50 μM only significantly increased the SOD activity, while the CAT activity was markedly enhanced only by the 50 μM NA and NR treatments (*p* < 0.05).

### 3.5. Anti-Inflammation Capacity of the Four NAD^+^ Precursors

**Figure 6:**
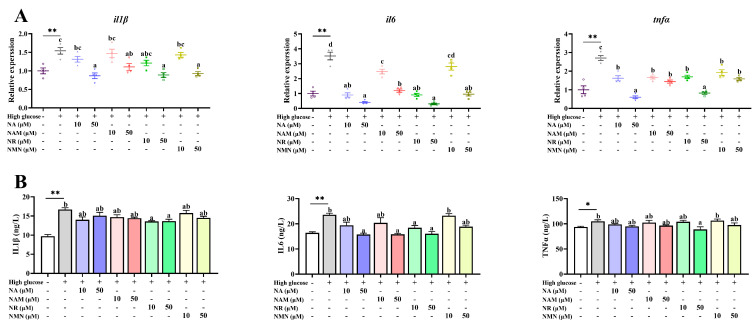
Effects of different NAD^+^ precursors on the transcription (**A**) and concentration (**B**) of inflammatory cytokines in the cell culture supernatants of primary hepatocytes exposed to a high-glucose medium (additional 60 mM glucose) for 48 h. Data (*n* = 4) are presented as mean ± SE. The asterisk (*) means a significant difference between the control and the high-glucose group (* *p* < 0.05, ** *p* < 0.01, *t*-test). Different letters mean significant differences among the high-glucose group and the group supplemented with NAD^+^ precursors (*p* < 0.05, Tukey’s test).

Compared with the control, the high-glucose incubation significantly up-regulated the gene expressions and concentrations of inflammatory cytokines, including IL1β, IL6, and TNFα in cell culture supernatants (*p* < 0.05). Only NR administration significantly decreased the IL1β and TNFα concentrations (*p* < 0.05) compared with the high-glucose group, while the IL6 concentration was obviously reduced by the treatments of 10 μM NR as well as 50 μM NA, NAM, and NR (*p* < 0.05) (Figure 6).

### 3.6. Anti-Apoptosis Capacity of the Four NAD^+^ Precursors

**Figure 7:**
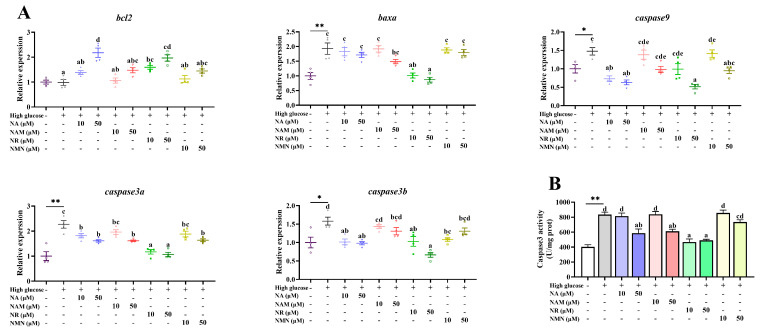
Effects of different NAD^+^ precursors on the transcription of apoptosis-related genes (**A**) and the caspase 3 activity (**B**) of primary hepatocytes exposed to a high-glucose medium (additional 60 mM glucose) for 48 h. Data (*n* = 4) are presented as mean ± SE. The asterisk (*) means a significant difference between the control and the high-glucose group (* *p* < 0.05, ** *p* < 0.01, *t*-test). Different letters mean significant differences among the high-glucose group and the groups supplemented with NAD^+^ precursors (*p* < 0.05, Tukey’s test).

As presented in Figure 7, compared with the control, the transcriptions of *baxa, caspase9, caspase3a*, and *caspase3b*, as well as the caspase3 activity, were all significantly increased by the high-glucose incubation (*p* < 0.05). Compared with the high-glucose group, the supplementation of 10 μM NR as well as 50 μM NA and NR markedly up-regulated the transcription of *bcl2* (*p* < 0.05). Moreover, the transcription of *baxa* was significantly down-regulated by NR addition, while that of *caspase 9* was remarkably decreased by treatments of 10 μM NA as well as 50 μM NA, NR, and NMN (*p* < 0.05). All NAD^+^ precursors reduced the transcriptions of *caspase3a* and *caspase3b,* with the lowest levels observed in the NR groups (*p* < 0.05). Furthermore, the caspase3 activity was significantly reduced by the treatments of 10 μM NR, as well as 50 μM NA, NAM, and NR (*p* < 0.05).

### 3.7. NAD^+^ Precursors Benefited Glucose Metabolism

**Figure 8:**
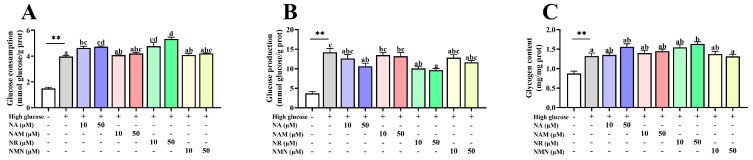
Effects of different NAD^+^ precursors on the glucose consumption (**A**) and production (**B**) and glycogen content (**C**) of primary hepatocytes exposed to a high-glucose medium (additional 60 mM glucose) for 48 h. Data (*n* = 4) are presented as mean ± SE. The asterisk (*) means a significant difference between the control and the high-glucose group (** *p* < 0.01, *t*-test). Different letters mean significant differences among the high-glucose group and the groups supplemented with NAD^+^ precursors (*p* < 0.05, Tukey’s test).

As shown in Figure 8, the high-glucose treatment significantly increased the values of glucose consumption, production, and glycogen content of hepatocyte (*p* < 0.05) compared with the control. Compared to the high-glucose group, NA and NR administration significantly increased glucose consumption, while the supplementation of 10 μM NR and 50 μM of NA and NR markedly decreased glucose production, and 50 μM NR supplementation markedly increased glycogen content (*p* < 0.05).

**Figure 9:**
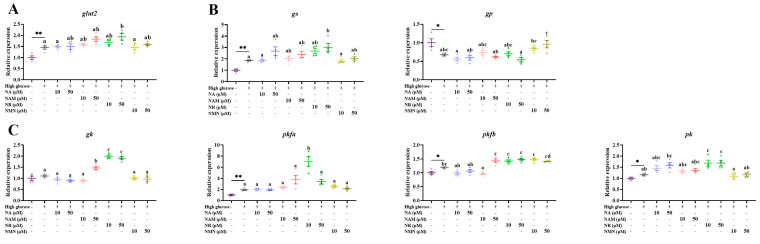
Effects of different NAD^+^ precursors on the transcription of glucose-transport- (**A**), glycogen-metabolism- (**B**), and glycolysis- (**C**) related genes of primary hepatocytes exposed to a high-glucose medium (additional 60 mM glucose) for 48 h. Data (*n* = 4) are presented as mean ± SE. The asterisk (*) means a significant difference between the control and the high-glucose group (* *p* < 0.05, ** *p* < 0.01, *t*-test). Different letters mean significant differences among the high-glucose group and the groups supplemented with NAD^+^ precursors (*p* < 0.05, Tukey’s test).

**Figure 10:**
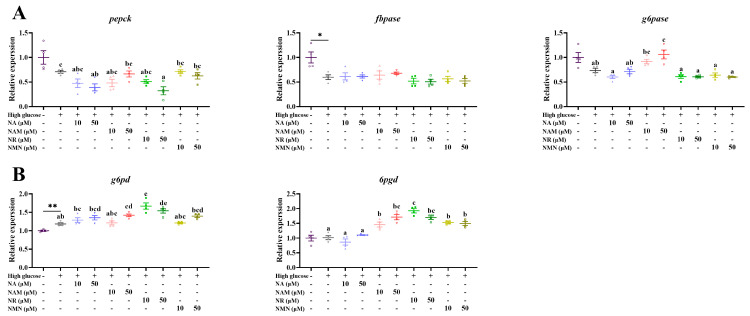
Effects of different NAD^+^ precursors on the transcription of gluconeogenesis- (**A**) and the pentose phosphate pathway- (**B**) related genes of primary hepatocytes exposed to a high-glucose medium (additional 60 mM glucose) for 48 h. Data (*n* = 4) are presented as mean ± SE. The asterisk (*) means a significant difference between the control and the high-glucose group (* *p* < 0.05, ** *p* < 0.01, *t*-test). Different letters mean significant differences among the high-glucose group and the groups supplemented with NAD^+^ precursors (*p* < 0.05, Tukey’s test).

As presented in Figure 9 and Figure 10, compared with the control, the transcriptions of *glut2*, *gs*, *pkfa*, *pkfb*, *pk*, and *g6pd* were significantly up-regulated, while that of *gp* was markedly down-regulated (*p* < 0.05). Compared with the high-glucose group, 50 μM NR supplementation markedly increased the transcriptions of *glut2* and *gs* (*p* < 0.05). The transcriptions of *gk* and *g6pd* were up-regulated by the treatments of 50 μM NAM, as well as 10 and 50 μM NR. The addition of 10 μM NR significantly increased *pkfa* expression, while 10 and 50 μM NR both up-regulated *pk* expression. The administration of 50 μM NA and NR both decreased the transcription of *pepck*, while that of *6gpd* was increased by NAM, NR, and NMN additions (*p* < 0.05).

### 3.8. The Oral Gavage Test Showed That NR Had a Superior NAD^+^ Promotion Effect In Vivo

**Figure 11:**
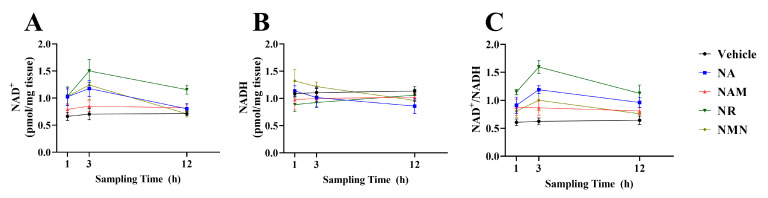
Hepatic NAD^+^ (**A**) and NADH (**B**) concentrations and the NAD^+^/NADH ratio (**C**) of fish after the oral administration of different NAD^+^ precursors. Data (*n* = 4) were presented as mean ± SE.

As shown in Figure 11 and Table 2, in terms of sampling time, the NAD^+^/NADH ratio at 3 h was significantly higher than those at 1 and 12 h (*p* < 0.05), while the NAD^+^ concentration at 3 h was only markedly higher than that at 12 h (*p* < 0.05). In terms of oral gavage, the NR group exhibited the highest NAD^+^ content among all the groups, revealing markedly higher values than the vehicle and NAM groups (*p* < 0.05). In addition, the NAD^+^/NADH ratio of the NR group was significantly higher than that of the other groups (*p* < 0.05).

## 4. Discussion

In the present study, an in vitro high-glucose model was successfully constructed in fish to mimic the liver injury induced by high carbohydrate intake. The high-glucose incubation decreased cell viability and increased transaminase activity at the same time, evidencing the emergence of hepatocytes injury. Meanwhile, decreased NAD^+^ content and NAD^+^/NADH ratio were also observed, as was in line with a previous study investigating the effects of high-carbohydrate feeding on the glycolipid metabolism of *M. amblycephala* [24]. Next, the effectiveness of four NAD^+^ precursors was compared in alleviating the high-glucose-induced hepatocytes injury. Strikingly, the supplementation of all four NAD^+^ precursors presented positive effects on the cell viability of hepatocytes exposed to high glucose, while NR showed the most profound effect. Furthermore, only NR significantly decreased the ALT and AST activities at the lower dose (10 μM). This indicates that NR may confer the most marked protection against the high-glucose-induced hepatocytes damage.

In the present study, the supplementation of NA exhibited a relatively low NAD^+^ boosting capacity compared with NAM, NR, and NMN, which might be due to the fact that the formation of NAD^+^ from NA needs more reaction steps. The NR-supplemented groups obtained higher NAD^+^ concentrations than NAM-treated groups, which may imply that the activity of nicotinamide riboside kinases is higher than that of nicotinamide phosphoribosyltransferase in fish. Moreover, NMN supplementation exhibited a lower NAD^+^ boosting capacity than NR. A possible explanation for this is that NMN cannot directly cross the cell membrane due to the lack of specific transporters [34]. Indeed, a previous study has proposed that NMN needs to be transformed into NR in the extracellular space before penetrating the cell membranes [35].

In the present study, NR administration increased the protein abundance of Sirt 1/3 compared with the high-glucose group, implying that NR might be most effective in regulating glucose metabolism and reducing oxidative stress among all four NAD^+^ precursors. This is supported by the facts that (1) both Sirt1 and Sirt3 are NAD^+^-dependent enzymes; (2) Sirt1 could regulate the activities of several transcription factors, which could consequently regulate glucose metabolism [36]; (3) while Sirt3 is related to the anti-oxidative defense of mitochondria [36]. The Sirt1/3 activation effects of NR have been attributed to be the enhancement of NAD^+^ formation via the Salvage pathway [11,37,38].

It is well accepted that oxidative stress is closely involved in high-glucose-induced organ damage, while NAD^+^ is an important participant in the regulation of redox balance [11,12]. In the present study, the MDA level of hepatocytes was exaggerated by high-glucose treatment coupled with decreased activities of SOD and CAT, indicating the occurrence of the dysfunction of the antioxidant system. Similar results have also been reported in the liver of *M. amblycephala* fed a high-carbohydrate diet [39], supporting the reliability of the in vitro high-glucose model in the present study. In addition, the supplementation of 50 μM NR markedly increased the activities of SOD, CAT, and GPX compared with the high-glucose group, while the treatment of 50 μM NA enhanced the activities of SOD and CAT, demonstrating that both NR and NA could activate the enzymatic antioxidant system. Notably, only the 50 μM NR group significantly reduced the MDA content, suggesting that NR displayed the most potent anti-oxidative property among four NAD^+^ precursors. This beneficial effect may be ascribed to the decreased mitochondrial ROS formation via Sirt3 activation since mitochondria are the main sites of cellular ROS generation, while Sirt3-mediated anti-oxidative enzyme activation is one of the most important components of mitochondrial anti-oxidative defense [5,40]. In the present study, the NR treatment up-regulated the Sirt3 abundance, which further supported this.

IL1β, IL6, and TNFα are cytokines that are well identified as the inflammation markers involved in the metabolic disease of fish [23]. In this study, the increased transcriptions and concentrations of IL1β, IL6, and TNFα were found in the hepatocytes exposed to high glucose compared with the control, suggesting that high-glucose treatment resulted in inflammation. The inflammatory response is usually exaggerated as a consequence of oxidative stress, which would worsen organ damage through apoptosis activation [41,42]. Under oxidative stress, the key pro-inflammatory regulator, namely nuclear factor kappa B (NF-κB), could be activated by overproduced ROS, thereby stimulating the inflammatory cascade via promoting the gene transcription of inflammatory cytokines [43]. A recent in vivo study in *M. amblycephala* has illustrated that high-carbohydrate feeding could activate the NF-κB pathway [23]. Therefore, it was presumed that the high-glucose-induced inflammation in the present study was partly mediated by ROS-induced NF-κB activation. Moreover, only NR simultaneously reduced the transcriptions and concentrations of IL1β and IL6 at a lower level (10 μM). The markedly decreased TNFα concentration was only observed in the group supplemented with 50 μM NR. The above results indicate that NR exhibits the best anti-inflammatory potency among the four NAD^+^ precursors in this study. This beneficial effect could be partly attributed to the antioxidant effect of NR due to the close relation between oxidative stress and inflammation. Furthermore, it should be noted that Sirt1 could directly suppress the NF-κB signaling through deacetylation [44]. Hence, the mechanism of the anti-inflammatory effects of NR may involve both Sirt1 and Sirt3 activation.

Oxidative stress and inflammatory actions are reported as strong inducers of the endogenous apoptotic pathway [45]. In the present study, the high-glucose treatment markedly up-regulated the transcriptions of *baxa, caspase9*, and *caspase3a/b* compared with the control. Cellular caspase3 activity was also enhanced by the high-glucose medium. In addition, only NR simultaneously up-regulated the transcription of the anti-apoptosis gene, *bcl2*, down-regulated the proapoptotic genes, *baxa* and *caspase 3a/b*, and reduced the caspase3 activity at the dose of 10 μM. Bcl2-associated X protein (Bax) family members are pivotal regulators of endogenous apoptosis. Among them, *bax* is a key proapoptotic gene, while *bcl2* is found to play an opposite role [46]. Furthermore, caspase 3 is the executioner of endogenous apoptosis, with its activity directly regulated by caspase 9 [47]. Therefore, the results obtained from the current study demonstrate that high-glucose treatment resulted in hepatocyte apoptosis, as was in line with a previous high-carbohydrate feeding study [23]. Meanwhile, NR has the most potent anti-apoptotic effect, which further emphasizes the most outstanding hepatocyte protection effects of NR among the four NAD^+^ precursors. The mechanism behind the anti-apoptotic effect of NR is likely multifaceted. Firstly, the attenuated oxidative stress and inflammation may help to inhibit apoptosis. Secondly, NAD^+^ acts as a substrate for poly (ADP-ribose) polymerases (PARPs), which play important roles in DNA repair [48]. NR administration increased cellular NAD^+^ concentration, which could consequently increase the PARPs activity, thereby exerting the anti-apoptotic effect by alleviating genomic DNA damage [49]. Thirdly, Sirt3 could improve mitochondrial health, which could decrease the release of proapoptotic factors from mitochondria [5]. Therefore, the activation of Sirt3 may also promote the anti-apoptotic effect of NR.

Multiple NAD^+^ precursors have been found to have favorable effects on glucose control [11,14]. However, limited evidence is available regarding the comparison of the effectiveness of different NAD^+^ precursors. In the present study, the hepatocytes under high-glucose treatment had higher glucose consumption amounts and glycogen contents compared with the control, which might be an adaptive response to improve glucose utilization. This is supported by previous in vivo studies, where the high-carbohydrate feeding up-regulated the transcriptions of the genes involved in glucose transportation, glycolysis, and the pentose phosphate pathway [36]. Moreover, the glucose-production capacity was also up-regulated by high glucose. This is not surprising given the fact that fish often maintain active gluconeogenesis even in the presence of sufficient exogenous glucose [50,51]. This is actually an important manifestation of glucose metabolism dysregulation in fish. Furthermore, compared to the high-glucose group, increased glucose consumption and glycogen content, as well as decreased glucose production amount, were observed in all NAD^+^ precursors groups, but to various degrees. Notably, the 50 μM NR treatment exhibited the highest glucose consumption level and cellular glycogen content, as well as the lowest glucose production. Furthermore, this treatment also resulted in the highest transcriptions of glucose transportation gene, *glut2*; glycogen synthesis gene, *gs*; glycolysis genes, *gk, pfka,* and *pk*; and the pentose phosphate pathway gene, *6pgd*. A down-regulation of gluconeogenesis genes *pepck* and *g6pase* was also observed in the NR-administrated group. These findings indicate that NR exhibits the most up-regulatory effect on glucose metabolism among the four NAD^+^ precursors. Generally, NR may influence glucose metabolism in multiple manners as a NAD^+^ precursor. On the one hand, NAD^+^ directly participates in glycolysis, the citric acid cycle, and oxidative phosphorylation as an important electron carrier. Therefore, the enhanced NAD^+^ level would activate these processes, thereby regulating glucose metabolism [10,14]. On the other hand, the elevated NAD^+^ level could activate Sirt1, which could modulate glucose metabolism pathways at the gene-expression level by regulating the related transcription factors involved in glucose metabolism [11,14].

The above results demonstrate that NR is the most effective NAD^+^ precursor to alleviate the high-glucose-induced hepatocytes injury. To further validate the in vitro results, an in vivo test was undertaken. As expected, the oral gavage of the four NAD^+^ precursors all increased hepatic NAD^+^ contents and the NAD^+^/NADH ratio with the highest values obtained in the NR treatment. Similarly, a previous study has indicated that NR administration exhibits a greater capability of NAD^+^ enhancement than NA and NAM in a mammal model [16]. Furthermore, the NAD^+^ boosting effect of NR is also reported in *Caenorhabditis elegan*s and *Drosophila melanogaster* [52]. These results indicate that the cellular NAD^+^-boosting effect of NR might be highly conserved among lower and higher animals.

## 5. Conclusions

In summary, the present study compared the effectiveness of four NAD^+^ precursors (NA, NAM, NR, and NMN) in alleviating high-glucose-induced hepatocyte damage in a fish model. The four NAD^+^ precursors all revealed beneficial effects, while NR exhibited the most potent effect in terms of cell viability, NAD^+^ boosting, oxidative defense, anti-inflammation, and anti-apoptosis. Moreover, NR could effectively regulate glucose metabolism by activating glucose transportation, glycolysis, glycogen synthesis, and the pentose phosphate pathway, as well as inhibiting gluconeogenesis at the transcriptional level. The above beneficial effects of NR can be partly attributed to the activation of Sirt1/3.

## Figures and Tables

**Table 1:** Primers used in the present study.

Gene Abbreviations	Gene Full Names	Primer Sequences (5′-3′)	Accession Numbers
*il6*	*interleukin-6*	ACAAAGCGCTCTTCCTGTTTG	KJ755058.1
		GCCATTTCTCCTGGTCGTTCA	
*il1β*	*interleukin-1β*	CGAAGGCATGTCGGAGCATT	XM_048181166.1
		ACCACTTCCATACGACGCTC	
*tnfα*	*tumor necrosis factor-α*	GCATGCCAGTCAGGTAGTGT	KU976426.1
		AGGGCCACAGAAAGAAGAGC	
*bcl2*	*b-cell lymphoma-2*	GATGAGCCCGTTAGTGGGAC	XM_048179299.1
		TCTGCGAATCGCTCCCATC	
*baxa*	*bcl2 associated X, apoptosis regulator a*	TCCTATTTTGGCACCCCCAC	XM_048196672.1
		CTCTCTGCTCCCCCTCATCT	
*caspase9*	-	TCCAGATGAGGTGGAACCCT	KM604705.1
		CCAAAATGTCGCTGGGTGTG	
*caspase3a*	-	GGAGCCTGACAGCCATAACA	KY006115.1
		TGAGCTCTAGTTGGTTGCCA	
*caspase3b*	-	TGGTATGTGCATGGGGAACA	XM_048187987.1
		TATGTGCATGGGGAACAGGAC	
*glut2*	*glucose transporter 2*	ACGCACCCGATGTGAAAGT	KC513421.2
		TTGGACAGCAGCATTGATT	
*gs*	*glycogen synthase*	CCTCCAGTAACAACTCACAACA	XM_048154697.1
		CAGATAGATTGGTGGTTACGC	
*gp*	*glycogen phosphorylase*	CTGTCTACCAGCTGGGGTTG	XM_048205686.1
		GGCCTTCTCCCAAGGGTTAC	
*gk*	*glucokinase*	ACTGGATCTTGGAGGGACGA	KJ141202.1
		AAGTCAGATATGCACCCGGC	
*pfka*	*phosphofructokinase a*	AGGAAATTGCAGTGCAGTAAAG	XM_048194728.1
		CTGCTTCTGCTTCTAAATCCGC	
*pfkb*	*phosphofructokinase b*	GAAACCGGCTCAGTCGAAGA	XM_048172371.1
		ACGGTGTAAACCCTGTGACC	
*pk*	*pyruvate kinase*	GCCGAGAAAGTCTTCATCGCACAG	XM_048152870.1
		CGTCCAGAACCGCATTAGCCAC	
*pepck*	*phosphoenolpyruvatecarboxykinase*	CGGCTACAACTTCGGTCAGT	XM_048198716.1
		ACGTGGAAGATCTTGGGCAG	
*fbpase*	*fructose-1,6-bisphosphatase*	TACCCAGATGTCACAGAAT	KJ743995.1
		CACTCATACAACAGCCTCA	
*g6pase*	*glucose-6-phosphatas*	CAGGCATGATTGTTGCCGAG	XM_048171060.1
		AATGGACCCAGGCTGGATTG	
*g6pd*	*glucose-6-phosphate dehydrogenase*	AGGTAAAGGTGCTGAAGT	KJ743994.1
		AAATGTAGCCTGAGTGGA	
*6pgd*	*6-phosphogluconate dehydrogenase*	TCAAGGAAGCGTTTGACCGA	XM_048178257.1
		CACTGTCATCTGTCAGGCGT	
*ef1α*	*elongation factor 1α*	CTTCTCAGGCTGACTGTGC	XM_048180512.1
		CCGCTAGCATTACCCTCC	

**Table 2:** Two-way ANOVA analysis of the results obtained from the oral gavage test. Means of main effects followed by the same superscript letter (lower case letters (a, b, c) for oral gavage and upper case letters (A, B)) in the same column are not significantly different (*p* > 0.05).

	NAD^+^ (pmol/mg Tissue)	NADH (pmol/mg Tissue)	NAD^+^/NADH Ratio
Means of main effects			
Oral gavage			
Vehicle	0.693 ^a^	1.110	0.626 ^a^
NA	1.001 ^ab^	1.005	1.022 ^b^
NAM	0.822 ^a^	0.999	0.852 ^ab^
NR	1.230 ^b^	0.957	1.291 ^c^
NMN	0.994 ^ab^	1.168	0.858 ^ab^
Sampling time			
1 h	0.909 ^AB^	1.082	0.871 ^A^
3 h	1.095 ^B^	1.054	1.057 ^B^
12 h	0.840 ^A^	1.008	0.861 ^A^
*p*-values			
Oral gavage	<0.001	0.186	<0.001
Sampling time	0.014	0.620	0.012
Interaction	0.378	0.459	0.448

## Data Availability

The data presented in this study are available on request from the corresponding author.
